# Movement perceived as chores or a source of joy: a phenomenological-hermeneutic study of physical activity and health

**DOI:** 10.1080/17482631.2018.1516088

**Published:** 2018-09-07

**Authors:** Sanne Angel

**Affiliations:** a Section for Nursing, Institute of Public Health, Aarhus University, Aarhus C, Denmark; b Faculty of Health Sciences and Social Care Molde University College, Norway

**Keywords:** Low back pain, heart disease, narration, rehabilitation, physical activity, phenomology-hermeneutic, Ricoeur, spinal cord injury

## Abstract

Physical activity has become the most documented and acknowledged health advice in relation to both staying healthy and regaining health both physically and mentally. Thus, physical activity in relation to spinal cord injury, low back pain and heart disease is respectively means to regain bodily function, avoid or reduce pain and early death.

A second analysis of three studies with a phenomenological-hermeneutic approach building on Ricoeur’s philosophy on how people understand themselves and their world through narrative configurations revealed that physical activity had different meanings to people. This revealed that the meanings of physical activity could range from movements being unpleasant, maybe even painful to movements being a source of joy. This caused participants (1) to engage in movement as a source of joy, (2) to overcome the bodily struggle to do their chores, and maybe feel better as a result or (3) to minimize bodily functions equivalent to a functional daily life. Illustrated by 10 different approaches this provides knowledge about driving forces for health professional support. As joy and passion are the strongest driving forces to physical activity, this highlights the importance of supporting people to find a kind of physical activity that they like.

## Introduction

During my studies of experiences among people with spinal cord injury, I was once asked, “what makes having a spinal cord injury such a tragedy?.” In my effort to give a clear answer I said that the core was loss of bodily functioning. Reflecting on my early years as a female nurse caring for patients with spinal cord injury at a neuro-intensive care unit, I realized that I mourned the patients’ loss of their lively, pulsating, communicating, joyous body that did as it was told. At the same time, I appreciated my own strong and flexible body that took me where I wanted. I could rely on my body and throw myself into anything being assured that my body would do as it was told. Moreover, the development of my body’s capacity to perform dressage and handle horses was an important project in my life.

**Table I.** The meaning of physical activity.

**1) To engage in movement as a source of joy**
The approach of a sportsman
The approach of a competitor
The approach of a social person
**2) To overcome the bodily struggle to do their chores, and maybe feel better afterwards**
The approach of an investor in health
The approach of a moral person
The approach of a conscientious person
The approach of a busy person
**3) To minimize bodily functions equivalent to a functional daily life**
The approach of a physically inactive person
The approach of a lazy person
The approach of an exhausted person

Revealing my personal reflection as a starting point, improvement of physique and physical capacity implies a continuous potential. Not only can the body continue to improve in relation to sports, we also rely on improvement when ill. This illustrates how bodily functioning is central in peoples’ world and is mirrored in the nursing researchers Thomas and Polio’s (2002 p. 89) study of pain addressing the body as the instrument of mastery over the world. However, when being ill or sustaining an injury, the challenge is if being in the world changes from “I can” to “I cannot.” Being a body is also to be limited by the possibilities of a body. Having and being a body able to move, the body becomes a source of freedom and independency; opposite, the body not being able to move become a prison and cause dependency (Thomas & Polio, 2002).

Training and physical activity play a major role (CDC, 2016; Pedersen & Saltin, 2015) and have become the most documented and acknowledged health advice to stay healthy and regain health both physically and mentally (Das & Horton, 2016). Physical activity in relation to spinal cord injury, low back pain and heart disease (as examples) is both a way to regain bodily functions, avoid or reduce pain and lower risk of early death. Research in rehabilitation after heart disease shows that the benefits are achieved by stronger muscle conditioning and improved cardio-respiratory fitness to obtain a healthier body both physically and mentally (Anderson et al., 2016).

The clinical problem in health care is the relation between illness and lack of physical activity. Caspersen, Powell, and Chistenson (1985) distinguish between physical activity, exercise, and physical fitness. Inspired by their work, physical activity in this paper is defined as movements made with the intention to improve or maintain physical fitness in relation to rehabilitation and maintenance of health. The fact that illness can be avoided and the consequences reduced by physical activity makes it a professional goal to make patients increase their level of physical activity. However, this may be challenging, especially that patients maintain the necessary level of physical activity as early research has shown (Laitakari, Vuori, & Oja, 1996).

A world review on nutrition emphasize that health professionals’ aim to “stimulate national governments to maximize their efforts to develop programs that encourage proper nutrition and participation in sports activities” (Simopoulos, 2004, p. XIV) highlights the importance. The lack of physical activity may partly be explained by everyday life in the Western World implying sedentary work and transportation. This means that physical activity that challenges the body is not necessary to accomplish daily tasks; it is a choice each person has to make. Combined with a lifestyle characterized by busyness, physical activity is often given a low priority. Thus, life does not have to entail physical activity beyond walking from one room to another, a few meters to and from the parking lot, moving when bathing, dressing, cooking and cleaning. These activities do not push the body physically. People thus may be physically active at a minimum, which often implies poor physical fitness (Hallal et al., 2012).

When recovering from injury and illness, physical activity plays a significant role. This is also illustrated in the recognized biopsychosocial International Classification of Functioning (ICF) model (WHO, 2013). For example, for a person with spinal cord injury, physical training to optimize the function of the neurologically damaged body is a central part of rehabilitation (Angel, Kirkevold, & Pedersen, 2009). Achieving the best possible physical functioning increases societal participation and reduces the need for compensation and changes in life.

However, the role of physical activity may be threefold as it both contributes to disease prevention, treatment/recovery, and health promotion. In persons with chronic heart disease, the physical activity is intended to modify biological cardiovascular risk factors. Often heart diseases are considered to be lifestyle-related, and therefore the core of rehabilitation is lifestyle changes. A study (Knudsen, Laustsen, Petersen, & Angel, 2014) of 20 heart patients showed three major groups of adherence to the health professional recommendation: (1) considered prior lifestyle to be appropriate, maybe with minor adjustments, (2) acknowledged the need for incorporating lifestyle changes, which some managed and some did not. However, others (3) reconciled or felt guilty, when unable to manage change.

In people with low back pain physical activity aims at improving physical fitness both to keep the pain at bay, to recover, and to achieve a well-functioning everyday life. A randomized control trial (Jensen et al., 2011) showed a significant reduction of low back pain, when exercising three times a week for 45 minutes. The intervention was based on the underlying assumption that previous experiences with physical activity could be decisive. If they had been physically active before it was assumed that it was easier to become physically active again. Having bodily knowledge, having an interest that could be rekindled, maybe even having been good at the activity, could cause joy in assuming the activity. However, the follow-up interview study (Angel et al., 2012) proved that people could succeed with taking up a new kind of physical activity; this was also the case in people who had never been physical activity before.

Thus, the goal of physical activity can be to recover, to be pain-free, to regain a well-functioning everyday life, and to survive. However, when these positive outcomes have been achieved, people may go back to doing a minimum of physical activity. The problem is that then the risk for a poor health increase (Angel et al., 2012). If people do not do the best for their health and survival this problem continues to be a health-related problem for which they are in need of help (Angel et al., 2012). To be supportive in relation to optimizing the performance of physical activity calls for knowledge of what physical activity means to each individual person. Physical activity promotion need to be responsive to the situational, motivational and meaning-related differences found in individuals as well as the particular health challenges that individuals face (Knudsen et al., 2014). The subjective valence or meaning of physical activity seems to be important to considered (Angel et al., 2012; Knudsen et al., 2014). Therefore, it may be quite important to know what “meanings” people assign to physical activity engagement, be it as a result of their own biographical experience, social norms or situational circumstances in order to tailor physical activity support effectively.

During 10 years of research in the field of recovery and rehabilitation, I have seen different ways of relating to physical activity. This paper reveals variations in the meanings physical activity had to people. The aim was to refine this insight to gain knowledge about the meanings physical activity can have to people and thereby support professionals to help people to be physically active.

## Method

This study is a second analysis (Heaton, 2004) of narrative interview from studies of three patient groups with different challenges. By reflecting on data from three studies, I tried to see, if there was more to learn. These same data were used to explore the meanings physical activity had on the participants. The new focus was narrower than the original research question that explored the experience of recovery and rehabilitation. This was possible due to the open approach to the data collection that allowed the participants to tell about their experiences.

All interviews used open-ended questions that encouraged the interviewees’ narrating. Using a phenomenological-hermeneutic approach, the meaning participants ascribed to physical activity was identified. The access to data from three studies provide a data material with rich variation. The narratives counted 11 participants with spinal cord injury (Angel et al., 2009) followed for 10 years, a study including a total of 40 participants with low back pain (Angel et al., 2012), and a study of 20 participants with heart disease (Knudsen et al., 2014). Ethical approval was achieved in relation to the primary studies (Angel et al., 2009; [no.2006-41-6190]; 2012 [no.2006-41-6190]; Knudsen et al., 2014; [no.2007-58-0010]) and all names are pseudonyms.

This development of knowledge built on Ricoeur’s (1983, 1985, 1988) philosophy on how people understand themselves and their world through narrative configurations. Therefore, participant’s narratives were read to achieve knowledge of the meaning physical activity had to them from what they said and thereby spoke of (Ricoeur, 1976). Moving forth and back between data and interpretation, I revealed meanings in participants’ lives as well as referring to the world in general.

The phenomenological-hermeneutic approach was in line with the methods in the primary studies. According to Ricoeur (2007, p. 26), “*phenomenology remains the unsurpassable presupposition of hermeneutics*.” and “cannot constitute itself without a *hermeneutical presupposition*.” This highlights that the initial interest in the meaning of a phenomenon can only come about due to a question for meaning, which in itself is grounded in hermeneutic (Ricoeur, 2007, p. 38). In this study, it is the need to know more about the meaning physical activity has to people. This arises from the presupposition that the meaning of physical activity will affect how people deal with the recommendation of exercising more. The investigation is made with a phenomenological approach in the effort to follow the meaning offered in the interview situation and later in the transcription of the interview. This implies that the researcher’s knowledge about the phenomenon is kept at bay in favour to the experience of the phenomenon. I did this by asking open question and facilitate the participant’s descriptions. The interviews were all conducted with this approach in the investigation of the experiences of recovery and rehabilitation.

The analysis consisted of naïve interpretation, structural analysis and critical interpretation. The naïve interpretation reflected my immediate impression and thereby my preunderstanding. Then the structural analysis was made reading sentence by sentence to cluster meaning units. In the structural analysis, the open phenomenological approach, looking for “what the text said,” letting the text show itself. However, in the process of understanding Ricoeur highlights the importance of explanation. “The hermeneutical condition of phenomenology is linked to the role of *Auslegung* [explanation] in the fulfillment of its phenomenological project” (Ricoeur 2007, p. 26). Reading “what the text talk about” does not add something new, but “in articulate and develop the experiences “to make it become itself” (2007, p. 39). The last part of the analysis is a critical interpretation, here the researcher through reflections find the most significant interpretation (Ricoeur, 1976). Thinking through the different meanings of physical activity represented, I recognized a deeper meaning also informed by literature (the most important is presented in the background), others and own experiences. This disclosed different experiences of physical activity from the meaning of physical activity as a chore to a source of joy.

## Findings

The first hurdle was whether physical activity was considered physical doable and manageable. Secondly, there seemed to be a major distinction between being motivated to be physically active by positive versus negative incentives. This implied that physical activity was experienced as a means to bodily well-being or a positive mood either during or after the activity, or a feeling of being satisfied with own actions. In contrast, physical activity could be experienced as a duty maybe even a chore motivated by fear and obligation. This implied a feeling of discomfort and maybe even pain. At best, there was a positive reward after the physical activity in the form of bodily well-being or good mood. But it could also imply, being unsure of whether this would lead to benefit in the form of well-being, or a positive self-image due to having done your duty or the recognition of being a dutiful or good person according to one self or others.

The analysis of the data revealed that physical activity can have different meanings for people. It can feel unpleasant, or even painful to some people, while it could be a source of joy for others. The way physical activity is thought about may also influence the way individuals actually engage in physical activity. Some seek to maximize enjoyment through regular and intense aerobic and motion exercises, others pursue physical activity as a means to an end in that it helps them to complete daily chores, while yet others restrict their physical energy expenditure to maintaining basic daily functioning. It is important to note that the meanings and related activity patterns can coexisted in individuals, depending on mood, situation or external motivators. Describing the meanings and related physical engagement as if they could be isolated is important to clarify their relative contribution in whether a person succeed in being physically active or not. The variations of the meanings the participants ascribed to physical activity could be demonstrated substantial in 10 approaches (Table I). Each may suggest as being activity motivated or demotivated by a particular set of factors, but in fact multiple meanings co-exist to a varying degree. For example, a person may be driven by the intention to do what is healthy, but at the same time feel to exhausted. The approaches were:

### To engage in movement as a source of joy

#### The approach of a sportsman

To the sportsman physical activity is both the mean and the goal. With the wish for and dedication to improvement, he is exercising being able to push himself beyond what feels good. The sportsman knows that improvement may course some pain. This enables him to be in the discomfort of the present enduring the body’s cry for a break.

Being a sportsman when having a spinal cord injury influenced Edward’s (participant with spinal cord injury in Angel et al., 2009) approach to rehabilitating his physical capacity. Like other persons with spinal cord injury he viewed the physiotherapy as the means to reduce the consequences of the injury and thereby a way to regain as much as possible of his life before the injury. Further, his knowledge of how to train was a basis for his participation and adding to the training. He expressed:

*“I’m a person who thinks that you can do something to achieve a goal.”*

*“..I have a will—a fighter will…if I get excited about something I can simply go on… and then I can do much more.”*

Being used to train he continued after discharge from the rehabilitation institution to contact highly qualified professionals to support him in continuing physical improvement. In his effort to get on with life, Edward was able to give it everything by exercising with diligence and consideration. The experience of the smooth function of the body becomes the driving force.

Edward was happy to get on a basketball team, and he could gather the courage to go more times a week to get the feeling of exercise and to have fun. Years later, an invitation to the wheelchair rugby team gave Edward an insight into the core of being a sportsman. Even though he had enjoyed basketball, this new sport suited his physical capacity better. Playing wheelchair rugby, he had a remarkable experience of being able to perform perfectly that made it possible for him to engage in sports at a new level. In basketball, he had never really reached top level due to his damaged shoulder; he did in wheelchair rugby. The experience of exhausting himself was ecstatic; it made him high for days and he dreamt of doing it again.

This provides an understanding of the transfer from joy of physical improvement to the spirit of physical excellence. The moments where being in your body is fantastic, all harmony, being one with yourself, weightless without being weightless. The performance, the movement, the body and mind all come together, like a perfect smash in tennis, like the swimmer moving smoothly through the water in total harmony and in dressage being one with the horse in such effortless movements. Thus, physical activity is for the sportsman a source of joy and satisfaction.

#### The approach of a competitor

Competition is a major part of sports; however, you may be a competitor without being a sportsman. To the competitor, physical activity is the means to the goal to win, and such a strong goal enables the person to push him/herself beyond limits where it hurts. This ability may be the reason why the person wins and it is the driving force. Competing is often related to competition being the best among others. However, this also implies competing towards the imaged other, which entails the ability to make a mental picture that enables the person to exceed his or hers limits.

From Elisa (participant with heart disease in Knudsen et al., 2014) in her seventies suffering from heart disease we learn about the competitor, when she explains her difficulties in being physically active, despite her belief that it would be good for her. Elisa describes not being a competitive person as a problem:

*“I have this problem that I’m not competitive at all. When I played badminton, I didn’t care at all if I win or lose. Then you don’t have this drive, you know.”*

Thus, we learn that when being a competitive person, performance matters and it is a driving force that makes the person go all in for physical activity. Competing is a way of being in the world like the racehorse energized alone by another horse being in front determined to take the lead. However, this energetic approach to life means that when no one else is available the competitor competes with himself.

#### The approach of a social person

To the social person physical activity in itself may not appear attractive. It may be considered directly unpleasant. However, even when disliking physical activity, the social person is encouraged to participate when invited by others. When convinced that being physically active is significant in relation to health, the social person searches for allies. To have appointments with others and looking forward to being with others is the driving force.

To Fin (participant with heart disease in Knudsen et al., 2014), who was in his late forties, the social dimension of his heart rehabilitation program played a crucial role. To him being part of a group meant that physical activity became a joy. Fin said:

*“Well it was, a damn joy going there…even though you were the youngest, and those people eh, in your team with you, they were rather old, the oldest was 88 dammit ..they were happy people ..and at the same time, you know, you could see every time you came out there that something had happened to them, and that must have happened to you too…so it was such a damn joy going there and meeting them, right.”*

Watching the others gave joy and with the joy Fin got more energy. Having insight into what drives you, you may be able to make arrangements ensuring to be physically active. However, after the heart rehabilitation program Fin did not continue exercising. Increasing in weight and being physically affected by his weak heart, he could not make himself join the team made by his former training group as planned.

### To overcome the bodily struggle to do their chores, and maybe feel better afterwards

#### The approach of an investor in health

Some people focus on health doing their best to do what is good for them. This approach may be inherited from family life, or by societal programs of health education. Physical activity is ascribed meaning in form of having a fit (maybe with focus on a beautiful) body, staying healthy, avoiding illness, and living a long life. In this way, physical activity becomes a kind of investment. This may be incorporated as part of a lifestyle and thereby not demand further considerations.

From time to time the investor in health has to reconsider physical activity. Getting out of shape due to age, illness and other changed circumstances calls for attention realizing that more should be done or changes should be made.

Living an active busy life and now suffering from low back pain, Jack (participant with low back in Angel et al., 2012) was called to attention at the age of 37 by a buddy:

*“If we should start to go out for a run and lose a few pounds…Then we have tried different types of training and then I found out that coming out and using my body in this way was also a tremendous help.”*

This revealed to him that he could improve his lifestyle aiming at being healthier. Even when living a healthy life, it has to be adjusted continually. This implied a continuously search for doing it better and adapting to the circumstances. Thus, physical activity was a mean to physical improvement and to be in physically good shape. The belief in doing something good for your body and mind was the driving force. This belief may be a significant source for feeling good during and/or after exercising.

#### The approach of a moral person

Similar to the person that invested in his or her health, the moral person considers physical activity the right thing to do. However, the incentive to be physically active differs. Even if the physical activity is perceived as a necessary evil, the personal satisfaction lies in being a person with a high standard and feeling morally good. This entails both internal and external evaluation. Dan (participant with heart disease in Knudsen et al., 2014) is in his seventies and told about his gratitude in relation to the health professional effort when he was rehabilitated after heart disease. Now it was up to him to make sure that their effort was not wasted. He told:

*“Well, it’s an amazing offer we get in this situation. Of course you have to, you have to deliver the goods back to the best of your ability….that’s for sure.”*

Thus, Dan did what he found to be the right thing due to the help received. In this case, the evaluator was Dan himself. This evaluation was related to a positive self-image. The external evaluator can also be at a very abstract level such as the health care professional, the society or God. The drive was the wish to do what is morally right to be a good person.

#### The approach of a conscientious person

To the conscientious person physical activity is perceived as a necessary evil. In contrast to the person investing in health and the moral person, the conscientious person considered it a duty, which implies being driven by obligation. This driving force differs from the moral person’s wish to be good according to standards of moral character. Instead, the conscientious person strives to avoid to be perceived as bad or unworthy by doing his or her duty.

Elisa (participant with heart disease in Knudsen et al., 2014) told how this kind of obligation had supported her to have an arrangement where she was expected to meet for exercising classes three times a week.

Elisa:
*“Yes, but if the agreement was that I would go there three times a week, then I do that, you know. When that arrangement was over, I had to do it [physical activity] three times a week myself.”*

Interviewer:
*“Yes.”*

Elisa:
*“but then I do that, right.”*

Elisa wished that this arrangement had continued as this was the way she could make it work. Some people, who acknowledge this fact, and sign up for such arrangements on a private basis. Thus, to do what you must do, this has to be to fulfil the expectations of others.

#### The approach of a busy person

To the busy person physical activity has a low priority due to a busy everyday program. Both job and family life entail many obligations without which life would not function. Everyday life may have too many tasks; getting up early in the morning forcing your not yet fully rested body to perform the first task of the day; waking up slowly while making lunch packages, making breakfast, waking up the children, helping, comforting, encouraging them to get dressed and ready, giving your partner a helping hand, too; continuing like this all day long and at the end of the day there is no time and energy left.

Ann, a working mother (participant with low back in Angel et al., 2012) to more children learned by a new episode with low back pain that she had to incorporate physical activities in her everyday life:

*“I can feel if I’m not doing my exercises two or three times a week. Then I start feeling tender in my lower back and think oh, what is wrong now. Well you have to exercise Ann. You have only been there once this week because you were busy.”*

Her focus was to overcome healing physical activities and get it done. For her a formalized exercise program after her children went to bed made it work.

The tasks and needs for leisure time in everyday life often do not leave the necessary time and resources for physical activity. Prioritizing physical activity demands the extra, which is difficult to mobilize without a certain incentive. However, even strong incentives concur with lack of resources to get it done. Even to the person that appreciates physical activity and sports they slide into the background when busy. To Andrew (participant with heart disease in Knudsen et al., 2014) the illness was the warning that called for reconsideration and change of life style. Andrew said:

*“I’ve made it a habit in normal weeks to drive home and be there around four every Tuesday and Thursday and then I get out bicycling.”*

The heart disease was the legitimate reason to reduce working hours and caused him to make room for his wish to go biking. Thus, for the busy person physical activity is manageable when being prioritized as a task among tasks.

### To minimize bodily functions equivalent to a functional daily life

#### The approach of a physically inactive person

To some people physical activity is not an integrated part of life. This may be due to culture, environment, or upbringing, and thereby interests and routines. Even strong incentives may not give raise to prioritize physical inactivity over other tasks. Bob (participant with heart disease in Knudsen et al., 2014) was in his seventies and an example of a person that was giving a low priority to physical activity. Despite, the numerous encouragements from his wife (who was a health professional) and doctors, he held on to his wish to focus on his company.

Bob:
*“Well I don’t know, it’s something to do with them saying that it should ideally be every day and I can feel that if you exercise every day then it’s good and it’s not always that I get it done every day”*

Interviewer:
*“No”*

Bob:
*“Well, when you’re out travelling or something or you’re busy doing things in the workshop or something like that.*

Interviewer:
*“Then time passes”*

Bob:
*“The time passes”*

Interviewer:
*“Yes”*

Bob:
*“Then you don’t bother.”*

This implied considering the time spent on physical activity would be at the expense of what he really wanted to accomplish.

Physical activity was not a part of Frank’s (participant with spinal cord injury in Angel et al., 2009) life either. Ten years after his spinal cord injury he had found a rhythm being able to do his job and having a satisfying everyday life. Being satisfied meant that he did not consider doing extra. Being physically active was to him accomplishing everyday tasks at home and at work.

To the inactive person, physical activity has a low priority and is only part of what is necessary to maintain everyday life activities. Thus, movements are only a means to accomplish everyday tasks. When these are done, the person keeps his body at rest. This lack of physical activity is not passivity because life can be lived without further physical strain. However, the core in physical inactivity may be that physical activity is experienced as too much strain feeling too uncomfortable and exhausting. Furthermore, the lack of interest may consequently induce a feeling of boredom and some people feel best when relaxing.

#### The approach of a lazy person

Everyone knows what it feels like to be lazy, when relaxing on the couch seems more important than the task you were supposed to perform. It may be questioned if it is the person or the body being lazy. The body may not respond when an inner dialogue on moving. This may be due to tiredness, heaviness and that it feels uncomfortable to move. Even the effort to force oneself may not succeed. Elisa (participant with heart disease in Knudsen et al., 2014) experienced it like that:

*“So even though I knew (…) that my fitness was not quite as it was when I finished the training, even though I knew I had to, I couldn’t at all and I’ve had an exercise bike and I don’t know, and then nothing really happened right, I go for a walk now and then but it’s not like my pulse is racing, you know.”*

It had been possible for Elisa to engage in the planned rehabilitation program. This showed that Elisa physically was able to do the activity. This also showed that by having a program, it became manageable for her; she could manage to join the program three times a week. Paradoxically, when she was supposed to do the exercises on her own she could not manage even though the activities were doable. The need for a program to overcome physical activities disclosed obligation as a possible driving force. Often this implies maintaining a strict routine and just missing once means that you are not able to get back into the routine.

#### The approach of an exhausted person

To some people physical activity is too demanding. Knowing all the benefits from being physically active, the exhausted person experiences movements as a strain and is physically inactive due to discomfort and pain. This person may not have experienced that the discomfort changes into a good bodily feeling or good results as the sportsman has. However, even the sportsman was about to lose his engagement when an improvement seemed to be impossible. Edward (participant with spinal cord injury in Angel et al., 2009) explained:

*“as soon as the progress stops (legs do not improve)…it is difficult to be optimistic”*

Then the expectation that the discomfort will slide into the background or stop cannot be the driving force to endure the unpleasantness experienced when being physically active.

Not being interested in physical activity, doing sports or being physically active is not a goal in itself. Then being active is something the person does with the aim of getting it over with. To the exhausted person none of the possible driving forces can conquer the exhaustion, which made sense to the other personality types.

The choice not to exercise despite recommendations was seen when physical activity was experienced as undoable. The question is whether it is a choice; when people, despite knowing the benefits, minimize physical activity the bodily strain is a major reason. This may rather be due to bodily issues like an unfit body, overweight, poor physical fitness, multiple diseases or disability. This, however, indicates difficulties in psychologically managing to get it done and manage to overcome oneself. Brian (participant with low back in Angel et al., 2012) was in his sixties and suffers from invalidating low back pain. He had almost given up.

Interviewer:
*You say that you ought to listen to the advice, so why don’t things change?*

Brian:
*“Well—it’s me—I must pull myself together and get started’’.*

Interviewer:
*‘‘Don’t you feel motivated?”*

Brian:
*“No, I am afraid not.”*

Interviewer:
*“What would it take to give you a push in the right direction?”*

Brian:
*“Oh, well—I have to get into shape—I have a little personal project going. I am losing weight. I must stop smoking because I am so out of shape. I am organizing deadlines for this. Then I will get hold of myself—the evenings are getting brighter—it is so awful going for a run when it is dark.”*

In cases like this the participant may feel encouraged by the prospects of a follow up meeting, not wanting to disappoint the health professional. This may indicate that professional support will work when everything else has not.

## Discussion

If people do not do what is best for their health and survival this has become a health issue of societal interest because a healthy population will increase wealth. Then the health professional anticipates that these people are in need of help. This weak paternalistic approach implies that health professionals urge people to do what the health professionals know is the best. When consulting health professionals wishing to feel good and be well, physical activity is often the recommendation. However, as seen in the studies (Angel et al., 2009, 2012; Knudsen et al., 2014) physical activity may not be the person’s own priority. Furthermore, the professional recommendations are not always doable and manageable. This calls for professionals that are able to address possible physical activity despite bodily limitations and peoples’ priority. This may be informed by possible driving forces like passion and joy, competing to reach a goal, doing the right things, doing what is good for you, or doing what you are obliged to. The presented approaches to physical activity illustrate possible driving forces that may be helpful in the dialog between the patient and the professional about how to be physically active.

The description of the different kinds of approaches may provide a fruitful basis for a patient-health professional diagnostic collaboration about the patient’s present situation and possibilities. E.g. being the lazy person (unable to do what you know is good for you) may be possible to change by the professional knowledge of how to suggest physical activities that are doable and manageable. This implies adjustment to bodily restrictions and practical limitations. Further, the person may be encouraged by fun, social engagement, a wish to be healthy, moral or duty. It may even evoke a competitive gene or a passion, which can be elaborated on.

The professional’s exploration of the possible driving force(s) implies dialogue with the patient. In this dialogue the professional conduct what Ricoeur (1983) names “followability” (p. 151). Hereby he highlights how following a person’s story is “… to understand the successive actions, thoughts and feelings” (Ricoeur, 1983, p. 150). This exploration calls for the patient’s exploration as well to be able to configure the story. This means that through the professional request for the patient’s story, the patient’s understanding of self and the situation increases. This happened during the patients telling about self in relation to prior physical activity, and in relation to the present situation to optimize future possibilities.

The different kinds of approaches address both intrinsic and extrinsic motivation, known from literature back to Harlow and colleagues’ learning experiment on apes in 1949. This is still applicable when explaining peoples’ actions acknowledging the stronger power in relation to maintenance of the intrinsic motivation coming from within the person themselves. Wigfield, Guthrie, Tonks, and Perencevich (2004) showed how this increased the students’ willingness to engage in tasks and improve their skills. Ryan and Deci (2000) explained that this builds on the significance of self-determination. The driving force revealed in the different approaches was a mixture of intrinsic and extrinsic motivations. These implied how the person understood the situation and him or herself and thereby also what he or she found possible This means that they offer the health professionals a deeper insight and thereby a larger repertoire of possible motivators. When health professionals for example recommend physical activity to people with health issues, the professionals represent an outer motivation. Even though the professional does not have the power to make people do as they say, people know what the professionals expect them to do. Thereby this serves as an extrinsic motivation that may be very helpful when the intrinsic motivation is suppressed by circumstances. This may be a desire to be recognition from others creating motivation (Ryan & Deci, 2000) in the form of a positive reinforcement. The motivation may be driven by a negative reinforcement in the form of an aim of getting rid of something—the pain, the bad conscience. Exploring the driving forces there can be a close relation between the intrinsic and the extrinsic motivation. For example, the sportsman’s urge to experience excellence may be related to the extrinsic motivation by being acknowledged for his performance. In the competitor, this may be even closer related because the energetic state of the competition is closely related to exceeding the others and win. However, Ryan and Deci (2000) stressed that if the extrinsic motivation gets higher than the intrinsic this has a negative effect on the experience of self-determination. This may have negative consequences because in relation to physical activity the experience of self-determination may be limited. Adhering to the health professional recommendations, physical activity may not seem as a choice. Rather it is presented as something everyone should do. However, the narrative representation of possible approaches may support the professionals go into dialog about the relationship between what the person does and who the person wants to be. This may address the persons’ self-determination.

Revealing different kinds of approaches through reflection on empirical data made it possible to disclose the surplus of meaning (Ricoeur, 1976) that data provided. Carefully and critically elaborating on the embedded content, the approaches appeared. That quotes from the same participants illustrated more than one approach support that they may coexist. This may be at different points in time or in a mix simultaneously. This provides more coherent understanding of the meaning of physical activity than when defined through predestinated categories like mental well-being, physical activity and weight status as in the work of Thøgersen-Ntoumani and Fox (2005). Still, extracting the different approaches is another way of exploring basic knowledge for interventions in physical activity than identifying narrative types of what exercise is to people as reported by Papathomas, Williams, and Smith (2015). They provided knowledge about people that actually did do exercise, and revealed that exercise could be experienced as restitution, medicine and redemption. The different approaches revealed the meaning of physical activity also when not doing the exercises as recommended.

### Limitations

This knowledge was developed from a larger group of people that had rehabilitated from three different diagnosis. This and the time span mean that the findings built on substantial data. In addition, the critical reflection on the upcoming meanings of physical activity went fort and back between findings, interpretations, and data, questioning if these meanings were the most significant interpretation (Ricoeur, 1976) or whether it could be otherwise. Despite the thorough analysis, I will not foreclose that meanings can be added. It may be during the use of this knowledge in clinical practice as well as in additional research. The latter is most needed in relation to patients suffering from severe health issues that make physical activity too much of a struggle.

### Implication for clinical practice

The important clinical contribution is that the professional must go into dialog with the patient about the meaning the patient assigns to physical activity. That the different meanings of physical activity may co-exist and change opens for that this may support the patient in disclosing a meaning that will make physical activity doable and manageable. The significance of a positive approach to physically active in relation to incorporating into daily life highlights the importance of supporting the patient in finding a physical activity that is experienced as joyful. If this is impossible, it may be fruitful to identify possible driving forces to establish meaning of physical activity to the individual. This may support the person to let discomfort slide into the background, and maybe exercise will improve bodily strength and skills and possibly paved the way for joy and passion.

## Conclusion

The meaning of physical activity differs among people and within people. The personal meaning of physical activity is decisive for how people respond to professional recommendations. Ten approaches revealed differences in the driving forces behind being physically active, which may inform health professionals’ recommendations on physical activity. Joy and passion were the strongest driving forces to physical activity. This highlights the importance of supporting people to find a kind of physical activity that they like. When movement is experienced as a chore, identification of the approach provides a deeper understanding and thereby a repertoire to find the individual’s driving force. The acknowledgement of the significance of a positive approach meant that this should be the end goal. This has consequences for maintenance of physical activity and thus maintenance of health and wellbeing.
